# Spinal Anesthesia Using the Double-Needle Technique for Thoracolumbar Spine Fracture Surgery: A Case Series

**DOI:** 10.7759/cureus.83288

**Published:** 2025-05-01

**Authors:** Richa Chandra, Carmine Pullano, Imran Ahmed Khan

**Affiliations:** 1 Anesthesiology, Rohilkhand Medical College and Hospital, Bareilly, IND; 2 Anesthesiology, Casa di Cura Privata Villa Salaria Hospital, Rome, ITA; 3 Community Medicine, KMC Medical College and Hospital, Maharajganj, IND

**Keywords:** double needle technique, hemodynamic stability, regional anesthesia, spinal anesthesia, thoracolumbar spine fracture

## Abstract

Spinal anesthesia (SA) has emerged as a viable alternative to general anesthesia (GA) for thoracolumbar spine surgeries. The double-needle technique (DNT) aims to enhance the precision and efficacy of SA in these procedures. The DNT (SA at two levels) involves performing two separate spinal punctures at different vertebral interspaces during the same procedure, often used to optimize anesthesia spread. This case series evaluates the feasibility, safety, and clinical outcomes of DNT in patients undergoing thoracolumbar spine fracture fixation. This case series includes five patients diagnosed with thoracolumbar spine fractures who underwent surgical fixation under SA using the DNT. Data on patient demographics, intraoperative hemodynamics, sensory and motor blockade characteristics, anesthesia-related complications, and postoperative recovery were collected and analyzed. All patients achieved adequate surgical anesthesia with the DNT. Hemodynamic stability was maintained in most cases, with minimal vasopressor requirements. None of the patients required conversion to GA. Multimodal analgesia was used to manage postoperative pain. No major anesthesia-related complications were observed. The DNT appears to be a safe and effective approach for SA in thoracolumbar spine surgery. It offers hemodynamic stability, adequate surgical anesthesia, and prolonged postoperative analgesia. Further studies with larger sample sizes are warranted to validate these findings.

## Introduction

The thoracolumbar region (T10-L2) is the most common site for spine fractures [1]. The comparatively rigid thoracic cavity and mobile lumbar spine make this area vulnerable to fractures [2]. Here, ribs are also not articulating anteriorly, i.e., floating ribs leading to a transition zone more prone to fractures. An additional anatomical factor contributing to vulnerability is that at this junction, the stiff kyphotic thoracic spine meets the caudad lordotic lumbar spine. The etiology remains falls from heights, motor vehicle accidents, recreational injuries, and work-related injuries [3]. These patients present with varying degrees of neurological deficits. These fractures are often associated with rib fractures leading to hemothorax or pneumothorax [4].

Spine fractures are traditionally operated on under general anesthesia (GA) due to concerns about patient positioning, airway management, and the difficulty in neuraxial block placement due to trauma [5]. Spinal anesthesia (SA) has been successfully utilized in spine surgeries due to various advantages [6]. Recent advancements in segmental SA, particularly thoracic segmental SA (TSSA), have demonstrated promising outcomes in select spine surgeries, offering advantages such as improved hemodynamic stability, reduced opioid requirement, early mobilization, and enhanced patient satisfaction [7,8].

However, achieving a consistent and reliable block for thoracolumbar spine procedures remains a challenge. A major limitation of conventional single-shot lumbar SA in thoracolumbar spine surgery is the regression of the sensory block. Typically, the block regresses in a cephalocaudal manner from higher to lower dermatomes over time, potentially compromising intraoperative anesthesia at the surgical site if we are using conventional lumbar SA. The double-needle technique (DNT) is a novel approach that involves sequential administration of different anesthetic agents at varying spinal levels to optimize the spread and duration of the block. This technique was originally reported during the COVID-19 era in an attempt to avoid GA in patients undergoing spine surgery for thoracolumbar spine fractures involving 39 patients [9]. This case series builds upon that experience by providing a detailed description of technical modifications and patient-centered outcomes in some select cases.

## Case presentation

**Figure 1:**
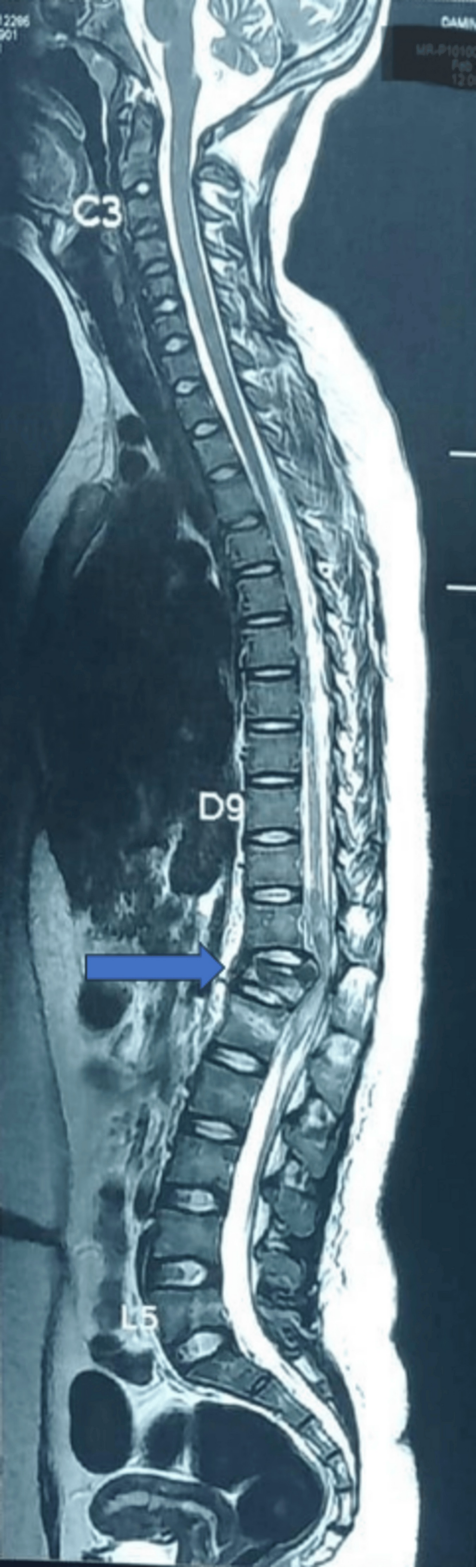
Sagittal MRI spine showing fracture at D12/L1, blue arrow (Case 3) MRI: magnetic resonance imaging; C: cervical; D: dorsal/thoracic; L: lumbar

We present a descriptive case series to evaluate the efficacy and safety of DNT for laminectomy and pedicle screw fixation of the thoracolumbar junction in five patients. All patients were informed about the risks and benefits of DNT, and prior informed consent was obtained. All patients agreed to allow the use of their data for publication, provided anonymity is maintained. The cases presented in this study were conducted at Rohilkhand Medical College and Hospital, Bareilly, between July 2024 and December 2024. The nature of the injury precluded the block placement in the center of the operative field. All patients had a history of falling from a height. Figure 1 shows a sagittal magnetic resonance imaging (MRI) scan of the spine showing a fracture at the D12/L1 spine level.

All five patients were male. Three patients had fractured ribs; one patient had an intercostal drainage (ICD) in situ for hemothorax. Four patients were smokers, and two patients had chronic obstructive pulmonary disease (COPD). Demographic and clinical characteristics of the participants are compiled in Table 1. All patients with spine trauma included in the study had varied levels of loss of sensory and motor function below the level of trauma.

**Table 1:** Demographic and clinical characteristics of the participants T: thoracic; L: lumbar; COPD: chronic obstructive pulmonary disease; HTN: hypertension; DM: diabetes mellitus; ICD: intercostal drainage

Participants’ characteristics	Case 1	Case 2	Case 3	Case 4	Case 5
Age (year)	67	80	45	52	48
Gender	Male	Male	Male	Male	Male
Weight (kg)	56	52	68	82	78
Diagnosis	T11–T12 fracture	T11 fracture	T12 fracture	T10 fracture	T12–L1 fracture
Smoker	Yes	Yes	Yes	Yes	No
Comorbidity	COPD	COPD, HTN	None	DM, HTN	None
Planned surgery	T10-L1 fixation	T10-T12 fixation	T11-L2 fixation	T9–T11 fixation	T11–L2 fixation
Associated injury	Fractured ribs with right-sided ICD	No associated injury	Fractured ribs	No associated injury	Fractured ribs

All patients were thoroughly informed about the novel nature of the technique, including its potential benefits and risks. Informed written consent was obtained from each patient after addressing all their queries. Furthermore, the technique was discussed in advance and agreed upon by the surgical team, ensuring a collaborative and ethically sound approach. A detailed preanesthetic checkup was done, and the patient was optimized. The patients were shifted to the operation theater after an overnight fast. A large-bore intravenous cannula was inserted on the dorsum of the hand, and Ringer's lactate was started. A pulse oximeter, a non-invasive blood pressure cuff, a five-lead electrocardiography, and a temperature probe were attached, and their functions were verified. All patients received an injection of ondansetron 8 mg and an injection of dexamethasone 8 mg before the start of the surgery.

All patients received SA using the DNT. The procedure was performed under strict aseptic conditions. Patients with spine fractures posted for surgery were positioned for SA by the operating theater staff, helped and guided by the operating surgeon and the anesthesiologist. The standard log-rolling maneuver was used to make them lateral.

**Figure 2:**
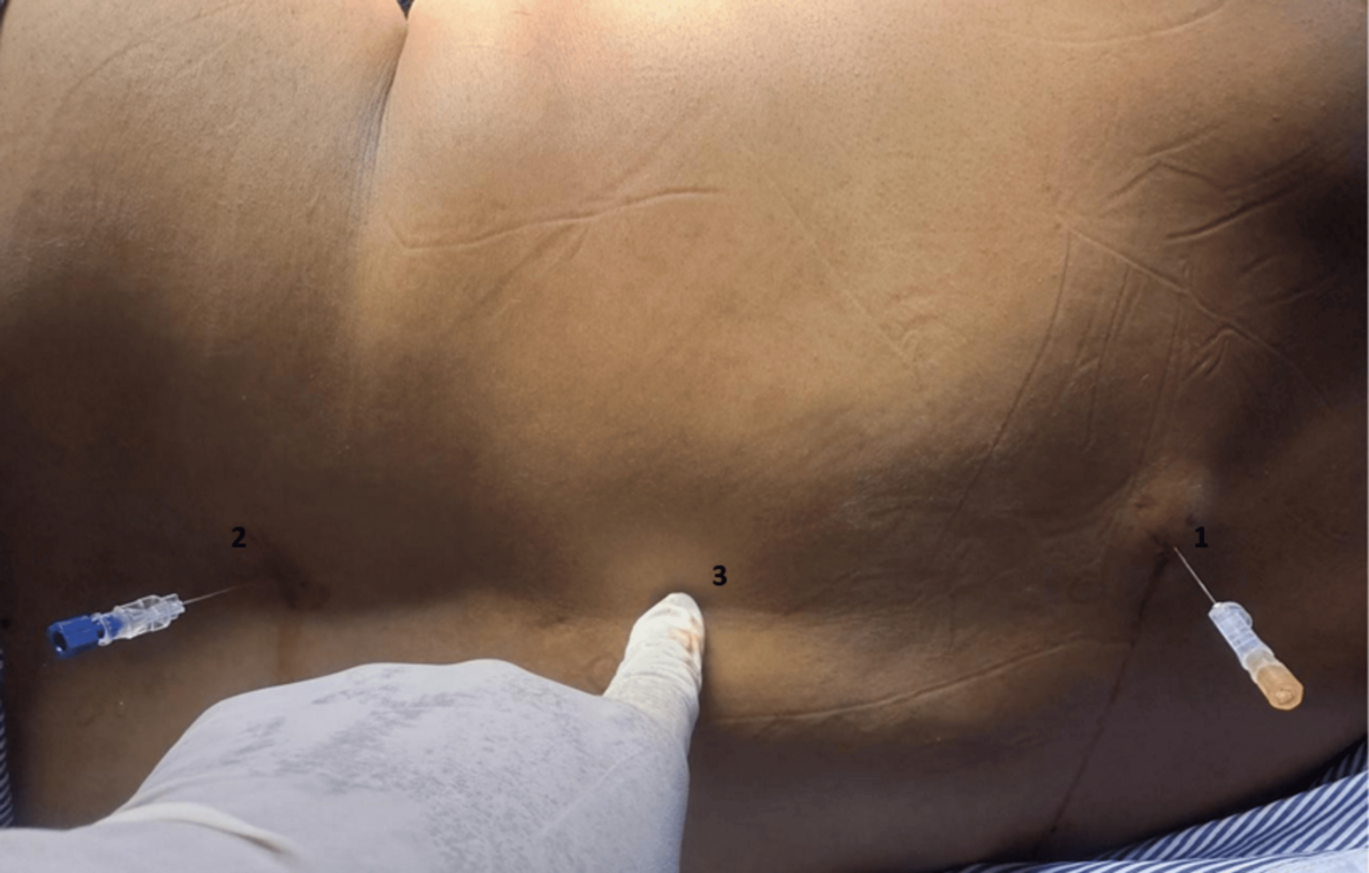
Spinal anesthesia at two levels (1 and 2) and fracture site (3)

SA was given at two levels using a Quincke spinal needle. The first injection was given at the T4-5/T5-6 level and the second at the L3-4/L2-3 levels (Figure 2). After putting in spinal needles and ascertaining clear CSF from both sides, the first injection with 1 mL isobaric levobupivacaine was given. The second injection at the lumbar level needs 1.5 mL isobaric levobupivacaine. Lower limb block was assessed by the Bromage scale, and for upper limbs, the epidural scoring scale for arm movements (ESSAM) was used [10]. ESSAM is a validated tool used to grade motor blocks following neuraxial anesthesia, particularly the degree of upper limb motor involvement. In our study, ESSAM was employed to objectively assess the extent and progression of motor blockade during and after the administration of the DNT.

The detailed technique of the DNT used in all cases is compiled in Table 2. We followed a predefined protocol to ensure consistency. Specifically, isobaric bupivacaine was administered in a dose of 5 mg via the first needle, followed by isobaric bupivacaine in a dose of 7.5 mg through the second needle, one after the other. Dexmedetomidine 5 μg was used in each syringe. Both syringes were labeled and prepared prior to the procedure to maintain clarity and reduce the risk of error.

**Table 2:** Anesthesia technique used among participants T: thoracic; L: lumbar; LA: local anesthetic

	Case 1	Case 2	Case 3	Case 4	Case 5
Position	Left lateral	Right lateral	Right lateral	Right lateral	Left lateral
First spinal given at	T5–6	T5–6	T5–6	T4–5	T5–6
Second spinal given at	L3–L4	L3–L4	L3–L4	L2–L3	L3–L4
LA	1 mL isobaric levobupivacaine at the upper puncture and 1.5 mL isobaric levobupivacaine at the lower puncture	1 mL isobaric levobupivacaine at the upper puncture and 1.5 mL isobaric levobupivacaine at the lower puncture	1 mL isobaric levobupivacaine at the upper puncture and 1.5 mL isobaric levobupivacaine at the lower puncture	1 mL isobaric levobupivacaine at the upper puncture and 1.5 mL isobaric levobupivacaine at the lower puncture	1 mL isobaric levobupivacaine at the upper puncture and 1.5 mL isobaric levobupivacaine at the lower puncture
Adjuvant	Dexmedetomidine 5 μg at both sides	Dexmedetomidine 5 μg at both sides	Dexmedetomidine 5 μg at both sides	Dexmedetomidine 5 μg at both sides	Dexmedetomidine 5 μg at both sides
Upper sensory block level	Up to T2	Up to T2	Up to T3	Up to T2	Up to T2
Intraoperative sedation	Butorphanol 1 mg	Nil	Butorphanol 1 mg	Nil	Nil

**Figure 3:**
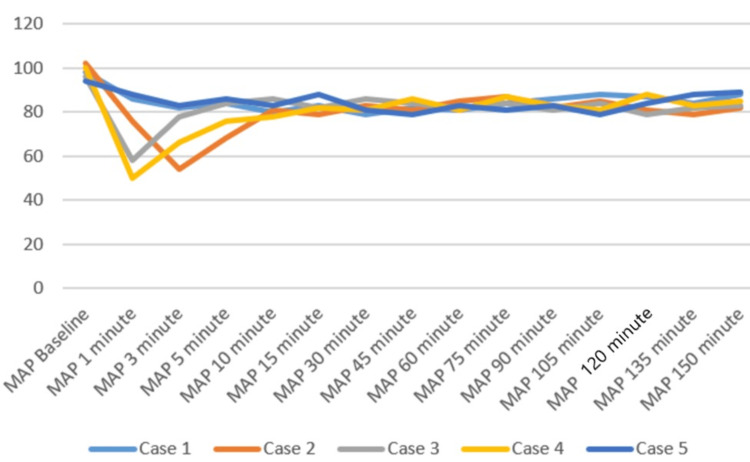
Mean arterial pressure (MAP, mmHg) of the participants over time

**Figure 4:**
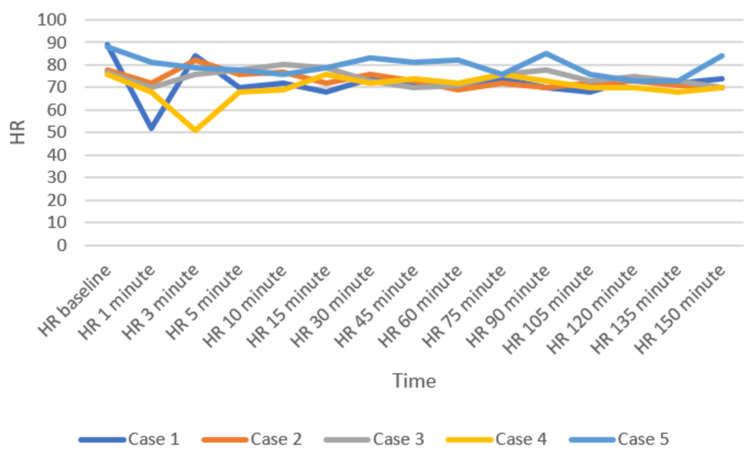
Heart rate (HR, per minute) of the participants over time

**Figure 5:**
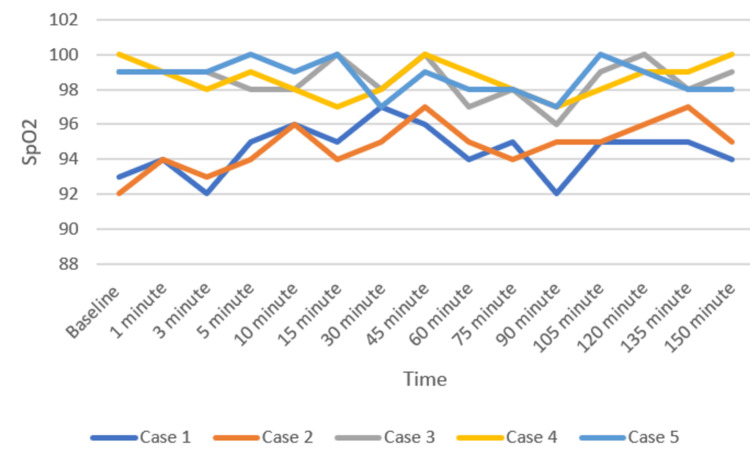
Peripheral oxygen saturation (SpO2, percentage) of the participants over time

Patients were positioned carefully in the prone operating position after ensuring adequate block effect. All patients were given a bilateral erector spinae plane block (ESPB) after prone positioning. One gram of tranexamic acid was injected intravenously prior to the incision. All patients were maintained on spontaneous respiration using Venturi masks with 2-4 L/min oxygen flow. The cut-off systolic blood pressure (SBP) for vasopressor use to label it as hypotension was a drop below 20% of the baseline SBP, or <90 mmHg, and a drop below 50/minute was labeled as bradycardia in the case series presented [11]. Vasopressor injection (mephentermine 6 mg intravenous bolus) and vagolytic injection (atropine 0.3 mg intravenous bolus) were provided as needed. Sensory and motor blockade characteristics, hemodynamic parameters (mean arterial pressure, heart rate), need for conversion to GA, postoperative analgesia duration, and adverse events (hypotension, bradycardia, nausea, and hypoxia) were monitored (Figures 3-5). All the patients have an ESSAM score of zero. The Bromage scale was grade 1 to 2.

Intraoperative complications are compiled in Table 3. A single episode of hypotension occurred in three patients, who responded to an injection of 6 mg mephentermine and a fluid bolus. Two patients required a single dose of injection of atropine 0.3 mg to manage bradycardia. Multimodal analgesia was used to tackle postoperative pain. No major complications like deterioration of neurological status in the postoperative period were observed. We could not study postdural puncture headache (PDPH), as our patients were in a lying position for a long time.

**Table 3:** Intraoperative complications among the cases PONV: postoperative nausea and vomiting

Intraoperative complications	Case 1	Case 2	Case 3	Case 4	Case 5
Hypotension	Nil	Present	Present	Present	Nil
Bradycardia	Present	Nil	Nil	Present	Nil
Paresthesia during spinal anesthesia	Nil	Nil	Nil	Nil	Nil
Respiratory difficulty	Nil	Nil	Nil	Nil	Nil
PONV	Nil	Nil	Nil	Nil	Nil
Bolster discomfort	Nil	Nil	Nil	Nil	Nil

## Discussion

This case series highlights the feasibility, safety, and efficacy of DNT in patients with spine fractures undergoing spine fixation surgery. The surgery duration was between 110 and 130 minutes with minimum blood loss. DNT may provide a practical alternative in patients with challenging spinal anatomy, including degenerative spine disease, scoliosis, or post-traumatic deformities, where standard single-site SA may be inadequate or unpredictable. By using two distinct intrathecal entry points, anesthesiologists can achieve more controlled and predictable sensory blockade [9,12]. However, larger studies are needed to confirm these preliminary observations and extrapolate this technique to other patient populations.

The nature of the injury frequently precluded the placement of the SA directly at the center of the operative field. This limitation necessitated careful selection of adjacent intervertebral spaces to ensure adequate anesthetic coverage while avoiding the manipulation of the surgical site, particularly in cases involving trauma or localized pathology. In this technique, the site of pathology and surrounding areas were excluded when administering SA, as many times this site contains a hematoma and is also intended for an implant. Therefore, we should keep our needles away from the site in this manner so that we can achieve the desired anesthesia at the pathology site.

The log-rolling maneuver was used without rolling, twisting, or curling of the spine [13]. In an average-sized adult patient, we need at least two persons, and in an above-average-sized adult, more persons are needed for the safe positioning of the patient.

Historical attempts in the development and application of the DNT have played a crucial role in shaping current clinical practices. These early efforts provided foundational insights into the anatomical feasibility, pharmacological considerations, and procedural safety of administering SA via dual entry points. In 1933, Fay and Gotten did punctures at two sites, one at cisterna magna and another at the lumbar level [14]. The thoracic approach of SA was reported by Imbelloni and Jonnesco. They gave SA for the skull, head, neck, and thorax at T1-T2 as the first puncture and the second puncture at the T12-L1 level. He found the middle thoracic puncture a difficult one [15]. Later on, myelographic studies showed ample space between the dura and spinal cord at the mid-thoracic level, making it safe for a subarachnoid block [16,17]. The DNT for SA has demonstrated feasibility and safety in thoracolumbar spine surgeries [9]. Compared to traditional single-injection SA, DNT allows for better control over sensory spread and duration of anesthesia. The stable intraoperative hemodynamics observed in our patients represent a notable advantage of the technique. Hypotension was observed in three patients and bradycardia in two patients, who responded quickly to a single dose of injection of mephentermine and atropine, respectively. A recent systematic review also concluded that hemodynamic instability occurs during TSSA, which is easily managed [18].

SA is considered to be a gold standard for awake surgery for the spine, especially when combined with any fascial plane block like the ESPB or thoracolumbar interfascial plane block, leading to shorter lengths of stay [19]. Fiani et al. analyzed 293 articles and concluded that awake spine surgeries can be done safely in those patients who cannot tolerate GA and in a mental state in which they can tolerate the prone position for the surgery [20].

Isobaric drugs are suitable because they are unaffected by position, and we have ample time for the second injection. We added adjuvant dexmedetomidine 5 μg at both places for the early onset and prolongation of the block [21].

All patients in our case series were difficult for GA, as weaning would have been an issue. Simple conventional lumbar SA would have covered the required dermatomes with a very large dose of local anesthetics and a head-down tilt. These patients were respiratory compromised and difficult to tolerate in a head-down position. This technique is very effective for tolerating bolsters because almost the whole thoracic spine is blocked and regression occurs from both sides, unlike conventional lumbar SA, where regression starts from above downward and tolerance of the upper bolster becomes difficult if the case is prolonged. It is more helpful if the patient is obese or female. The block remains for a variable time period according to the type and volume of adjuvant used.

Awake patients help themselves to make their position prone because a lower dose, i.e., 1 mL of local anesthetics at the mid-thoracic level, led to intact hand movements. All the patients have an ESSAM score of zero. This again prevents patients from any face, eye, neck, or brachial plexus injuries.

One gram of tranexamic acid was administered before incision in all cases. This has the added advantage of an already vasodilated field leading to less blood loss. Additionally, spontaneous respiration leading to less engorgement of epidural veins needs very little cautery and, again, less surgical time. This less blood loss is in favor of surgeries under regional anesthesia.

Another question arises about the need and requirement of sedation in these prone patients. Unlike other fractures, we do not use any type of splint or plaster of Paris casts prior to the definitive treatment, and these patients used to face excruciating pain constantly. Even a minor position change gives them a lot of pain. It is seen that after giving a block, these patients undergo sleep because of relief from pain. Only a few patients need a supplemental small dose of sedation in the form of butorphanol or midazolam. The authors recommend a sedated but arousable patient.

These patients have a typical contour of the back after getting a fracture in the middle part of the spine, so we recommend not injecting anything near the fracture site. Any injection away from the fracture site leads to non-interference with the surgical field and prevents the sharing of the same field for the placement of an implant.

Imbelloni found that higher blocks, e.g., thoracic spinal blocks, need lesser doses of intrathecal local anesthetics, leading to less hemodynamic instability and lower duration of sensory and motor blocks compared with using conventional high doses in the lumbar area [22]. Recently, Imbelloni and Chandra emphasized DNT in the journey of thoracic SA from the 20th century to the modern era [23]. Previous studies have suggested that SA can be safely performed using modified techniques [24,25]. At present, DNT cannot yet be considered a routine choice universally, but in our center, it has been selectively adopted in specific clinical scenarios where enhanced control and safety are desirable and GA poses more risk. Its use is based on institutional experience, preliminary evidence, and careful patient selection. DNT offers potential advantages such as better control over block height, segmental spread, and minimized hemodynamic fluctuations-factors particularly relevant in surgeries requiring selective anesthesia with stable physiology. Hypotension and bradycardia occurred in the initial few minutes, which responded to fluid boluses and a single shot of mephentermine and atropine.

This study has several limitations. The case series design and small sample size limit the generalizability of the findings, and the absence of a control group and statistical analysis restricts objective comparison with conventional techniques. All the cases were performed by experienced anesthesiologists in a high-volume center and, thus, may not be suitable for novice practitioners. Additionally, patient selection was non-randomized and specific to thoracolumbar fractures, which may not reflect broader clinical scenarios. Lastly, the reproducibility and long-term outcomes of the DNT remain to be validated through larger observational studies or randomized controlled trials.

## Conclusions

This case series highlights the potential benefits of the DNT in thoracolumbar spine surgery. The approach provided adequate surgical anesthesia, stable hemodynamics, and prolonged postoperative analgesia without significant complications. The present case series builds upon preliminary experiences to provide procedural refinement, practical applicability, and clinical outcomes that may inform broader clinical adoption. With standardized protocols and further research, this method can offer a valuable alternative to GA, especially in high-risk patients or resource-limited settings.

## References

[1] Aghakhani K, Kordrostami R, Memarian A, Asl ND, Zavareh FN (2018). The association between type of spine fracture and the mechanism of trauma: a useful tool for identifying mechanism of trauma on legal medicine field. J Forensic Leg Med.

[2] Bruno AG, Burkhart K, Allaire B, Anderson DE, Bouxsein ML (2017). Spinal loading patterns from biomechanical modeling explain the high incidence of vertebral fractures in the thoracolumbar region. J Bone Miner Res.

[3] Mittal S, Rana A, Ahuja K, Ifthekar S, Sarkar B, Kandwal P (2021). Pattern of spine fracture in sub-Himalayan region: a prospective study. J Clin Orthop Trauma.

[4] Spiegl UJ, Osterhoff G, Bula P, Hartmann F, Scheyerer MJ, Schnake KJ, Ullrich BW (2022). Concomitant injuries in patients with thoracic vertebral body fractures-a systematic literature review. Arch Orthop Trauma Surg.

[5] Wahood W, Yolcu Y, Alvi MA, Goyal A, Long TR, Bydon M (2019). Assessing the differences in outcomes between general and non-general anesthesia in spine surgery: results from a national registry. Clin Neurol Neurosurg.

[6] Garg B, Ahuja K, Sharan AD (2022). Regional anesthesia for spine surgery. J Am Acad Orthop Surg.

[7] Haloi P, Biswas R, Bora AK, Mahanta D, Choudhury D (2024). Thoracic segmental spinal anesthesia in kyphoplasty: a case series. Bali J Anesthesiol.

[8] Boykov N, Ferdinandov D, Vasileva P, Yankov D, Burev S, Tanova R (2024). Thoracic spinal anesthesia with intrathecal sedation for lower back surgery: a retrospective cohort study. Front Med (Lausanne).

[9] Chandra R, Pullano C, Misra G, Agrawal P, Singh A (2023). Double needle technique-a novel approach of anaesthesia for thoracolumbar spine fractures. Austin J Anesth Analg.

[10] Abd Elrazek E, Scott NB, Vohra A (1999). An epidural scoring scale for arm movements (ESSAM) in patients receiving high thoracic epidural analgesia for coronary artery bypass grafting. Anaesthesia.

[11] Ferré F, Martin C, Bosch L, Kurrek M, Lairez O, Minville V (2020). Control of spinal anesthesia-induced hypotension in adults. Local Reg Anesth.

[12] Fehlings MG, Alvi MA, Evaniew N (2024). A clinical practice guideline for prevention, diagnosis and management of intraoperative spinal cord injury: recommendations for use of intraoperative neuromonitoring and for the use of preoperative and intraoperative protocols for patients undergoing spine surgery. Global Spine J.

[13] Groeneveld A, McKenzie ML, Williams D (2001). Logrolling: establishing consistent practice. Orthop Nurs.

[14] Fay T, Gotten N (1933). Controlled spinal anesthesia: its value in establishing appropriate levels for chordotomy. Arch NeurPsych.

[15] Imbelloni LE (2010). JONNESCO: one century of thoracic spinal anesthesia history. Braz J Anesthesiol.

[16] Lee RA, van Zundert AA, Breedveld P, Wondergem JH, Peek D, Wieringa PA (2007). The anatomy of the thoracic spinal canal investigated with magnetic resonance imaging (MRI). Acta Anaesthesiol Belg.

[17] Chandra R, Misra G, Pokharia P, Singh PK (2024). Study of thoracic spinal canal in Indian population with the 3.0 Tesla magnetic resonance imaging: exploring the safety profile of thoracic spinal anesthesia. J Anesth Clin Res.

[18] Karim HM, Khan IA, Ayub A, Ahmed G (2024). Comparison of hemodynamic and recovery profile between segmental thoracic spinal and general anesthesia in upper abdominal and breast surgeries: a systematic review and meta-analysis. Cureus.

[19] Wilson JP, Bonin B, Quinones C, Kumbhare D, Guthikonda B, Hoang S (2024). Spinal anesthesia for awake spine surgery: a paradigm shift for enhanced recovery after surgery. J Clin Med.

[20] Fiani B, Reardon T, Selvage J (2021). Awake spine surgery: an eye-opening movement. Surg Neurol Int.

[21] Naaz S, Bandey J, Ozair E, Asghar A (2016). Optimal dose of intrathecal dexmedetomidine in lower abdominal surgeries in average Indian adult. J Clin Diagn Res.

[22] Imbelloni LE (2014). Spinal anesthesia for laparoscopic cholecystectomy: thoracic vs. lumbar technique. Saudi J Anaesth.

[23] Imbelloni LE, Chandra R (2025). The state of the art of thoracic spinal anesthesia. From Jonnesco in the early 20th century to the present day. J Anesth Crit Care Open Acces.

[24] Lee JK, Park JH, Hyun SJ, Hodel D, Hausmann ON (2021). Regional anesthesia for lumbar spine surgery: can it be a standard in the future?. Neurospine.

[25] Perez-Roman RJ, Govindarajan V, Bryant JP, Wang MY (2021). Spinal anesthesia in awake surgical procedures of the lumbar spine: a systematic review and meta-analysis of 3709 patients. Neurosurg Focus.

